# St. Louis College for Medical Practitioners

**Published:** 1882-05

**Authors:** 


PRELIMINARY NOTICE OF THE COLLEGE FOR MEDICAL PRACTITIONERS, ST. LOUIS. Inaugurated, 1882.

Object: To teach Medical Practitioners, by practical instruction, the special branches of medicine and surgery.

There will be twelve departments, so arranged that special courses may be taken with as little loss of time as possible.

Full particulars with regard to the time that special courses may be taken, the kind of clinics, the terms etc., will soon be ready.

The following gentlemen have been elected to fill a part of the departments:

Thos. F. Rumbold, M. D.; Ewd. F. Borck, M. D.; W. Hutson Ford, M. D.; Wm. Dickinson, M. D.; W. B. Outten, M. D.; Col. Fred. T. Ledergerber, Att'y. at Law; C. H. Hughes, M. D.;

Others who have had especial advantages in their departments will be added:

For particulars, enquire of the Dean, Dr. Thos. F. Rumbold, 1225 Washington Ave.; or the Secretary, Dr. Edw. Borck. N. E. Cor. of Fourth and Market Sts. St. Louis, Mo.
March 21, 1882.

